# Dancing to a different tune: changing reproductive seasonality in an introduced chital deer population

**DOI:** 10.1007/s00442-022-05232-6

**Published:** 2022-08-12

**Authors:** Catherine L. Kelly, Lin Schwarzkopf, Iain J. Gordon, Anthony Pople, David L. Kelly, Ben T. Hirsch

**Affiliations:** 1 Division of Tropical Environments and Societies, James Cook University, Townsville, Australia; 2 Fenner School of Environment and Society, Australian National University, Canberra, Australia; 3 James Hutton Institute, Craigiebuckler, Aberdeen, UK; 4 CSIRO, Australian Tropical Science and Innovation Precinct, Douglas Campus, Townsville, Australia; 5 Central Queensland University, Townsville, QLD Australia; 6 Department of Agriculture and Fisheries, Brisbane, QLD Australia; 7 Waikato Institute of Technology, Hamilton, New Zealand; 8 Smithsonian Tropical Research Institute, Panama, Panama

**Keywords:** Invasive species, Seasonality, Reproductive physiology, Reproductive skew, Deer

## Abstract

Male and female reproductive behaviour is typically synchronised. In species such as those in the family *Cervidae*, reproductive timing is often cued by photoperiod, although in females, it can be dependent on body condition. When a species is introduced to a novel environment, the environment changes, or responses of the sexes to such cues differ, asynchronous reproductive behaviour between males and females may occur. We investigated the seasonality of reproductive behaviour in introduced chital deer in northern Queensland by examining male antler phase in relation to female conception rates. We then analysed the influence of different variables likely to affect the timing of male and female reproductive physiology. The lowest percentage of chital in hard antler in any 1 month in this study was 35% (Fig. 1), but the average value was closer to 50%, thus there was a seasonal peak in antler phase linked with photoperiod. Females conceived at any time of year, but were strongly influenced by the amount of rainfall 3 months prior to conception. This resulted in varying conception peaks year-to-year that often did not correspond to the male’s peak in hard antler. In this system, a proportion of males and females were physiologically and behaviourally ready to mate at any time of the year. We predict that differences in the timing of the peaks between the males and females will lead to increased reproductive skew (variation in reproductive success among individual males). This pattern may select for different mating strategies or physiological mechanisms to increase reproductive success.

**Supplementary Information:**

The online version contains supplementary material available at 10.1007/s00442-022-05232-6.

**Fig. 1:**
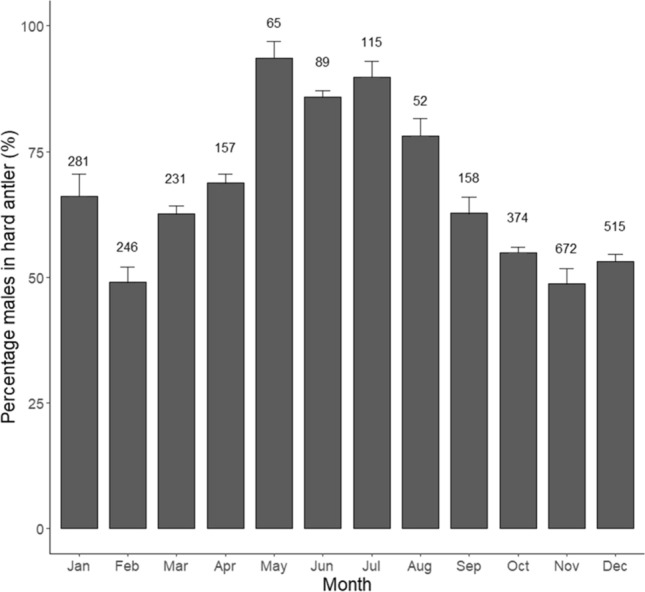
The average percentage of male chital deer in hard antler by month from 2014 to 2019 in north Queensland. Values above the bars indicate the total number of males that were sampled in each month and the error bars indicate the standard error. In the month with the lowest % males in hard antler in the entire study (November, 2017), 35% of males were in hard antler

## Introduction

Many species exhibit seasonal peaks in reproduction. Usually, the physiological reproductive cycles of males and females are triggered by the same environmental factors (O’Brien et al. 2003). In other species, male and female cycles are influenced by different mechanisms, but these events usually occur with sufficient predictability, or temporal synchrony, that male and female cycles match (Ball and Ketterson 2008). For example, female breeding cycles in many temperate birds are influenced by food availability, whereas male cycles are triggered by photoperiod (Moore et al. 2005). In these instances, male and female cycles remain synchronised, because days get longer in spring, and food availability also increases in spring (Moore et al. 2005).

When photoperiod does not vary strongly, which often occurs in tropical environments, the signals for reproductive timing may not be robust, which can lead to differences in the physiological breeding season between males and females (Spinage 1973; Bronson 1988; Moore et al. 2005). If the triggers for reproduction become temporally uncorrelated over evolutionary time, then we might expect linking mechanisms like sperm storage or embryonic diapause to evolve (Birkhead and Moller 1993). In species exposed to rapidly changing environments, due to climate change or being introduced to new environments, physiological reproductive cycles of males and females may become unsynchronised (Paoli et al. 2018). Here, we define this mismatch as temporal shifts in male and female breeding patterns. Although several studies have examined the causes of among-population asynchrony in male and female breeding cycles (Primack 1980; Post et al. 2001; Moore et al. 2005; Walter et al. 2015; Waddle et al. 2019), few have examined reproductive asynchrony between sexes within a population, to distinguish its causes, or in populations introduced into novel environments where seasonal cues could be different from the native environment.

Deer provide an excellent system to study asynchrony in reproductive periods. Deer exhibit wide among-population variation in physiological reproductive cycles, ranging from extremely synchronised (all reproduce at the same time) to completely asynchronous populations (reproduce throughout the year with no discernable peaks; Asher et al. 2000). For many deer species, the environmental cues affecting male and female reproductive cycles are well understood (Mitchell and Lincoln 1973; Bubenik et al. 1991; Clements et al. 2010; Rodriguez-Hidalgo et al. 2010; Asher 2011). Both male and female reproductive cycles may be triggered by changes in photoperiod (Lee 1970; Lincoln et al. 1984; Bubenik et al. 1987; Asher 2011), but the ability of females to come into oestrus is strongly influenced by conditions that support pregnancy, such as high food availability or mild weather (McGinnes and Downing 1977; Asher 2011). Female body condition can act as a threshold for reproduction, whereby body condition above this threshold will permit pregnancy (Bronson 2009; Clauss et al. 2021). As such, the degree of synchrony in female receptivity may be influenced by factors such as the length and severity of periods with low resources (Bronson 2009; Clauss et al. 2021).

In most deer, males undergo a synchronised annual cycle where antlers are grown and then shed (Bubenik et al. 1987; Tomas 1995; Ungerfeld et al. 2008). Antlers are used during confrontations with other males to secure mates, and act as an indication to females of male fitness and condition during a period called the rut (Bubenik et al. 1991; Clements et al. 2010; Vanpe et al. 2010; Heckeberg 2017). During this cycle, males that are without antlers, or still growing antlers (i.e., in velvet), are not competitive with stags in hard antler (Gosch and Fischer 1989). In many deer, this period of hard antler is also associated with an increase in testicular volume, and sperm quantity and quality (Lincoln et al. 1984; Loudon and Curlewis 1988; Gosch and Fischer 1989; Willard and Randel 2002; Hernandez-Souza et al. 2013). In some deer, males are unable to reproduce out of hard antler (Gosch and Fischer 1989; Goeritz et al. 2003), while others, such as chital deer (*Axis* *axis*), can produce viable sperm regardless of season and antler phase (Loudon and Curlewis 1988; Chapman and Harris 1991; Willard and Randel 2002).

Although ungulate breeding cycles have been extensively examined in relation to latitude (Spinage 1973; Fletcher 1974; Bubenik et al. 1990; Bonenfant et al. 2003; Loe et al. 2005; Asher 2011; English et al. 2012; Pereira et al. 2020), causes of within-population and between-sex asynchrony are seldom examined (Moyes et al. 2011). As few studies have examined reproductive synchrony between the sexes in introduced mammals, examining introduced deer allows comparisons of reproductive patterns in the same species exposed to environments with new or different factors affecting reproduction (Fletcher 1974; Bubenik et al. 1990; Asher 2011; Moyes et al. 2011).

Chital deer are a sub-tropical to tropical cervid native to India, Nepal, and Sri Lanka, that have been introduced to locations around the world including Argentina, Chile, Croatia, Hawai'i, Texas, and Australia (Long 2003). Male chital deer grow their antlers for approximately 5 months (velvet), and retain hard antlers for approximately 7 months, before shedding them and beginning the cycle again (Loudon and Curlewis 1988). In their native range, the presence of hard antlers in adult male chital is associated with lengthening photoperiod (Raman 1997; Sankar and Acharya 2004; Ramesh et al. 2012). While in hard antler, testes size, sperm volume, and sperm quality increase (Loudon and Curlewis 1988; Willard and Randel 2002). There is typically a seasonal peak in conceptions that correlates with peak rutting activity in males, and fawns are born during the winter dry season when fawn survival should be supported by high food availability (Graf and Nichols 1966; Mungall and Sheffield 1994; Ahrestani et al. 2012; Ramesh et al. 2012). It is believed that chital deer are capable of siring offspring while not in hard antler (Chapman and Harris 1991), and females may enter oestrus in any month of the year (Mylrea et al. 1999; Ahrestani et al. 2012). Despite this ability, males in hard antler would have a major competitive advantage, and males without antlers may not have the behavioural opportunity to mate with females (Graf and Nichols 1966).

In this study, we examined the synchrony of male and female chital reproduction in a population introduced to tropical Australia in 1886 (Roff 1960). We predicted that, as in their native and invaded ranges, male antler phase would show a seasonal peak (Willard and Randel 2002). Given the highly seasonal rainfall in northern Australia, and the influence of rainfall on chital body condition and abundance, we predicted that timing of female conceptions would be heavily influenced by rainfall patterns (Watter et al. 2019). Specifically, we predicted rainfall in the 3 months prior to conception would allow sufficient time for females to acquire the necessary condition to permit pregnancy. We predicted that if males exhibited seasonal peaks in hard antler, and rainfall is important to female reproductive phase, we may observe asynchrony between peaks in male hard antler phase and female conceptions.

## Materials and methods

To investigate patterns of antler growth in male chital deer, we collected data between 2014 and 2019 using camera traps, culls, and incidental observations. Camera traps were deployed at Spyglass Beef Research Facility, a cattle property covering 38,221 hectares in the Charters Towers region, North Queensland, Australia. Three seasons occur at the study site: wet (summer—January to March; 12.55 daylight hours), cool dry (winter—April to August; 11.07 daylight hours), and hot dry (spring—September to December; 12.52 daylight hours), although the amount and timing of rainfall in these seasons can vary greatly. This region is considered semi-arid, experiencing highly seasonal rainfall (average 689 mm; CV(annual rainfall) = 47%), with ~ 75% of the rainfall falling between November and March; Supplementary Material 1 Fig. 1).

Camera trapping was conducted with 94 Bushnell Aggressor cameras spaced at least 500 m apart (methods detailed in Supplementary Material 2). Cameras were set up approximately 30–50 cm above and perpendicular to the ground, and faced north or south to avoid the rising or setting sun. Cameras were deployed for at least 1 month each between October 2017 and November 2018. There were 3 months when cameras were not active (May–July 2018). Cameras captured three images per trigger, with a 1-s delay between photos. All photos were time- and date-stamped. Consecutive triggers of deer < 60 min apart were excluded from analysis to avoid pseudoreplication. Images were identified and organised using WildID software and ZSL CTap software (Amin et al. 2014; TEAM Network 2017 [https://www.wildlifeinsights.org/team-network]). Records of incidental observations of males were also collected between 2014 and 2019. In analyses of both camera trap images and incidental observations, only stags that could be positively identified were included. If a stag’s antler stage was uncertain or unknown, it was excluded. The total number of males that were sampled using camera traps and incidental observations was pooled per month, as well as the total number of males sampled that were in hard antler in each month. These values were used to calculate the monthly proportion of males that were in hard antler.

Female reproductive seasonality was determined by dissection of females culled from 2014 to 2019 in the Charters Towers region (20.0770°S, 146.2601°E). Chital were shot from a helicopter on nine properties in the region in October–November 2016 (five properties), November 2017 (three properties), and March 2018 (three properties) as part of a governmental feral animal control program. Research samples also were taken from animals shot on properties (10 males and 10 females on each occasion) in October 2014, March 2015, October 2015, and March 2016 (Watter et al. 2019). Because chital deer are legally declared a pest animal (Queensland’s Biosecurity Act 2014), no permits were required for culls on private lands. Deer shot for research were under the authorisation of the Queensland Department of Agriculture and Fisheries (Ethics permit number: SA 2014/07/475).

If a shot female was pregnant, the foetus was weighed. We calculated the age of foetuses using equations parameterised from prior data (Graf and Nichols 1966). Graf and Nichols (1966) reported a gestation length of 229 days, and a birth weight of 3690 g for chital (in Hawai'i, average female chital mass was 44.36 kg, and the average female mass in this study was very similar, at 45.0 kg). We used these values to develop age estimates based on foetal size at dissection. Chital foetuses grow exponentially for the first 120 days (when their mass is < 560 g), and then growth slows and becomes linear (Graf and Nichols 1966). We developed two equations to predict age: one for each growth phase, depending on foetus size at culling. For the initial exponential growth phase, we assumed a mass at day 1 of 1.1396 (g) (Graf and Nichols 1966), and a growth constant of 0.0504. For foetuses < 560 g (*R*^2^ = 0.986), we calculated age as:

$$\mathrm{age\,(in\,days}) =\mathrm{ln}(Y/1.3196)/0.0504,$$

where *Y* equals foetus mass (g). For foetuses larger than > 560 g, and in the linear growth phase (*R*^2^ ~ 1), we calculated:

$$\mathrm{age\,(in\,days})=(Y-2832.25)/27.95.$$

These equations allowed us to determine the date of conception for each pregnant female, by subtracting foetus age in days from the date of dissection. We determined conception date for 130 pregnant females from shot samples.

Conception dates were grouped into months to generate monthly conception numbers between 2014 and 2019. Because no cull was undertaken in early 2017, conception dates from December 2016 to March 2017 are missing.

### Analyses

Seasonal patterns of males in hard antler were tested using a cosinor model, estimated using the cosinor function in the R package “season”. The cosinor model tests for the presence of a sinusoidal component, which is an indication of seasonal patterns. The seasonality effect size is reported as the amplitude component of the model (Barnett et al. 2021). To investigate environmental variables that may influence male reproductive seasonality, we constructed generalised linear models with the total number of males in hard antler as the response variable, with the total number of males that were sampled per month included as an offset. As our analyses were based on counts (i.e., the number of males in hard antler per month), we used a Poisson distribution with a log link function. The average daylength (58 year average), year, monthly rainfall in the 0, 1, 3, and 6 months prior to a given month, as well as a year–rainfall interaction (to test for year-to-year differences) were used as predictor variables. Monthly rainfall totals for the region were obtained from the Australian Bureau of Meteorology for months between July 2013 and March 2018. Rainfall totals were calculated for periods 0, 1, 3, and 6 months prior to each month (including the month of each antler measure) and used as predictive variables. The best model was identified by the lowest AIC value using package *MuMIn* (Anderson et al. 2000; Barton 2019). Where a single top model could not be identified, i.e., there were multiple top models with a ΔAIC_c_ < 2, model averaging (the practice of using multiple models for making predictions; Banner and Higgs 2017) was performed and the full average results are presented (Burnham and Anderson 2002). If antler phase in males was related to photoperiod, we expected peak hard antler occurring in months with lengthening days as it is in the native range (Moe and Wegge 1994; Sankar and Acharya 2004; Umapathy et al. 2007). If male antler phase was influenced by resources, we expected a relationship between antler phenology and rainfall.

To examine whether conceptions were seasonal, we calculated the percentage of conceptions per month. These percentages were calculated as the number of conceptions that were observed in a particular month (determined using the above formulas) divided by the number of culled females that could have been pregnant during that time period, and multiplied by 100. Female seasonality was also tested using a cosinor model (Barnett et al. 2021). To determine the factors that may have influenced conception rates (the response variable), we constructed models using a range of biological and environmental variables as predictors. We used the total number of females that conceived in a month as the response variable, and the total number of females that could have conceived in each month was included as an offset to account for different sample sizes. As our analyses were based on counts (i.e., the number of females that conceived per month), we used a Poisson distribution with a log link function. Because conception in many deer species is related to resource availability, we used rainfall as a proxy for vegetation quality. As photoperiod or seasonal factors strongly influence the timing of reproduction in deer, we included average monthly absolute daylength (Jan 1993–Dec 2017). Finally, to investigate if conceptions were correlated with male antler phase, we included the proportion of males in hard antler (and therefore, presumably, the proportion of males in breeding condition). If males and females are reproductively synchronised, we would expect the presence of hard antlers to be positively correlated with conception rate. We used generalised linear models (using the *glm* function) to examine relationships between the proportion of monthly rates of conception and rainfall (0, 1, 3, and 6 months prior to conception), year, photoperiod, year and photoperiod interaction, and the proportion of males in hard antler. The most parsimonious models were again determined using AIC, and model averaging was performed if there was more than one top model (ΔAIC_c_ < 2). All analyses were conducted in R (V3.6.2, R Core Team 2019) and visualised using the *ggplot2* package (Wickham 2016).

## Results

**Fig. 2:**
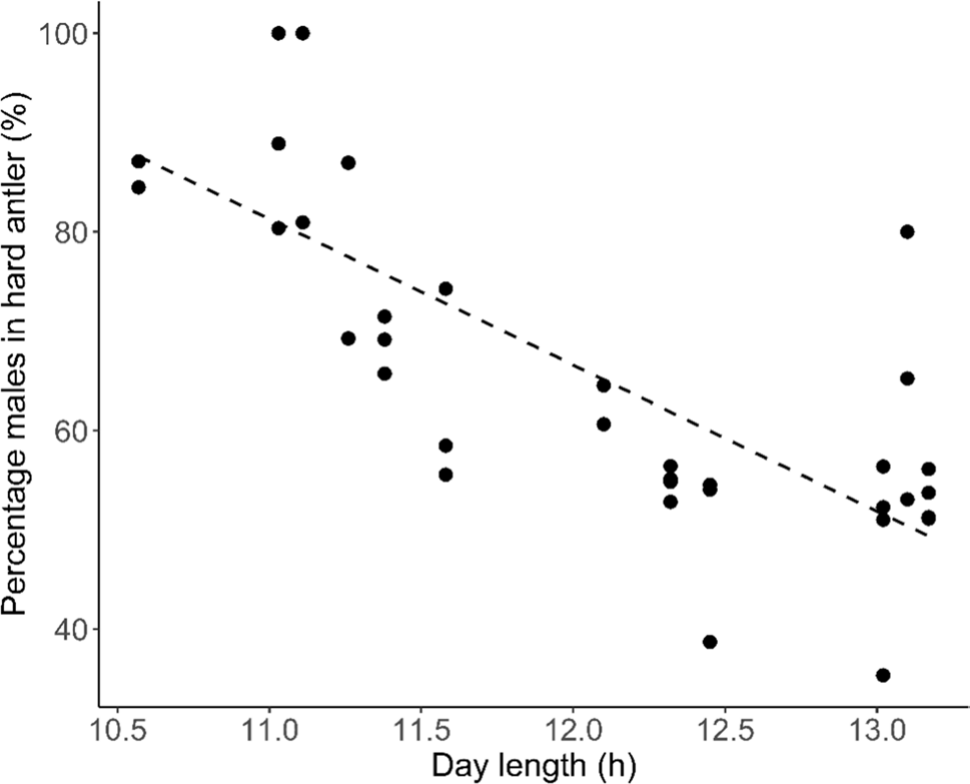
Relationship between the percentage of male chital deer in hard antler and absolute daylength (h) in the period 2014–2019 in north Queensland

We recorded the antler stage of 2239 stags in incidental observations (*n* = 1530) and photos from camera trapping (*n* = 709). Of these, we recorded 923 stags in hard antler (41.22%). The proportion of males in hard antler was highest from May–August (median month—5.8). While males exhibited a significantly seasonal pattern (Table 1), males in hard antler were observed year round, with almost 50% of males in hard antler even during the average monthly minimum in the study period (Fig. 1). Following model selection (Supplementary Material 1 Table 1), there were two top model candidates (daylength + 6 month rainfall (AICc = 0.00) and daylength + 0 month rainfall (AICc = 0.58), so model averaging was performed. Peak hard antler phase was best explained by absolute daylength only (Table 2), where shorter days correlated with more stags in hard antler (*R*^2^ = 0.570; Fig. 2). Models including other variables (i.e., rainfall, year, and photoperiod) were not supported. When we retested these hypotheses with temperature as a predictor instead of photoperiod (which were not tested together because they are highly correlated; *r* = 0.919), temperature was not supported.

**Table 1:** Parameter estimates of cosinor model examining the seasonal patterns of the proportion of male chital deer in hard antler (reproductively active) between 2013 and 2018

	Estimate	Std. error	*t* value	*p* value
(Intercept)	0.70	0.01	47.92	< 0.001
sinw	0.11	0.02	5.39	< 0.001

Significant sinw values indicate seasonal reproduction

**Table 2:** Model-averaged parameter estimates from the best (Δ AICc < 2) generalised linear models (GLM) from Supplementary Material Table 1 for the number of stags in hard antler in a given month with the total number that were sampled per month included as an offset

	Estimate	*z* value	2.50%	97.50%
Intercept	1.94	3.06	0.695	3.180
Daylight	− 0.20	4.13	− 0.299	− 0.107
6 month	0.00	2.65	0.001	0.001
0 month	0.00	2.54	0.000	0.002
1 month	0.00	2.50	0.001	0.001

**Fig. 3:**
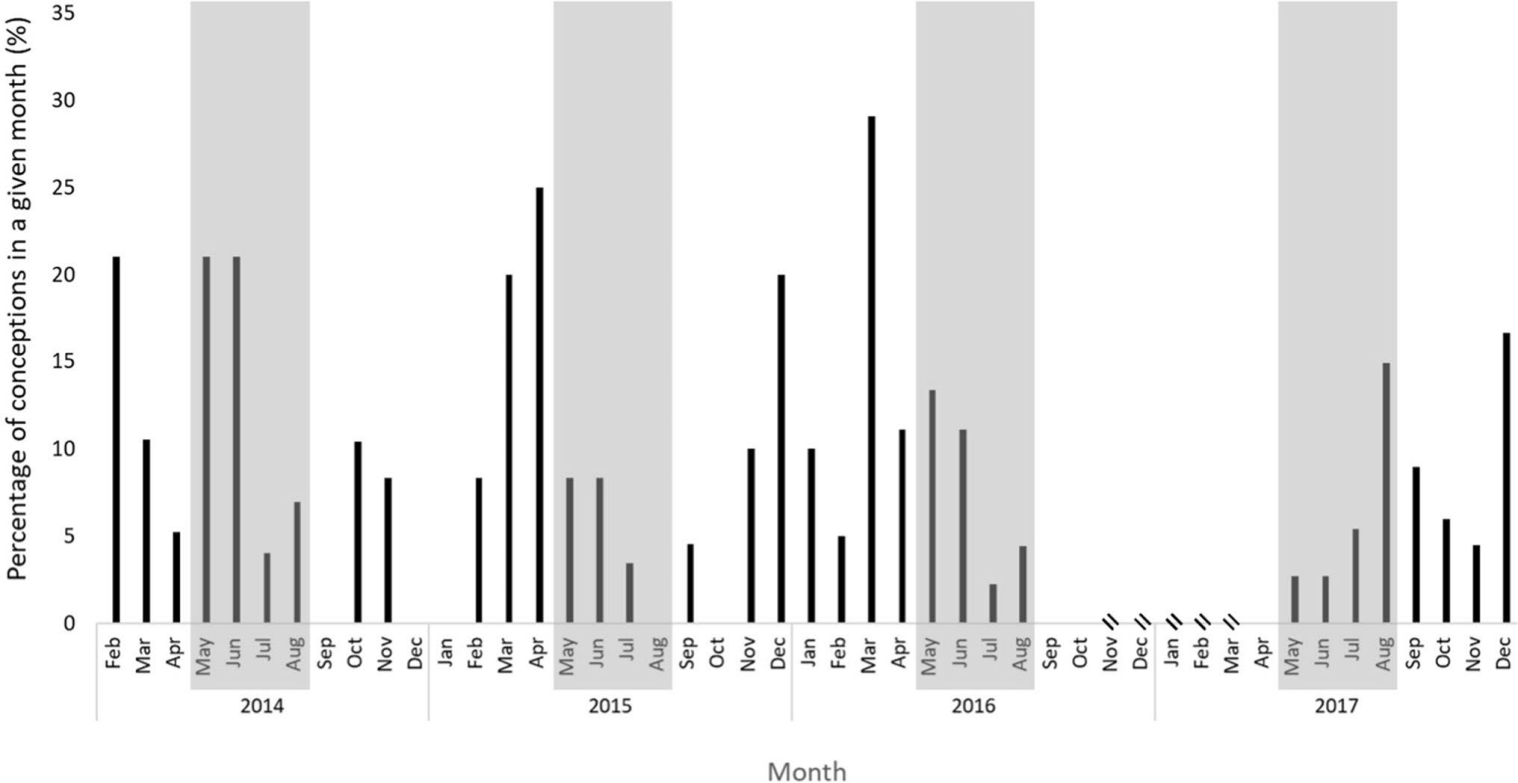
Percentage of females chital that conceived in any given month in the Charters Towers Region, Australia, over 2013–2018. Shaded regions represent the period during which males are in peak hard antler, which we predicted should be periods of peak conceptions if males and females are in synchrony. Double angled bars indicate no data

**Fig. 4:**
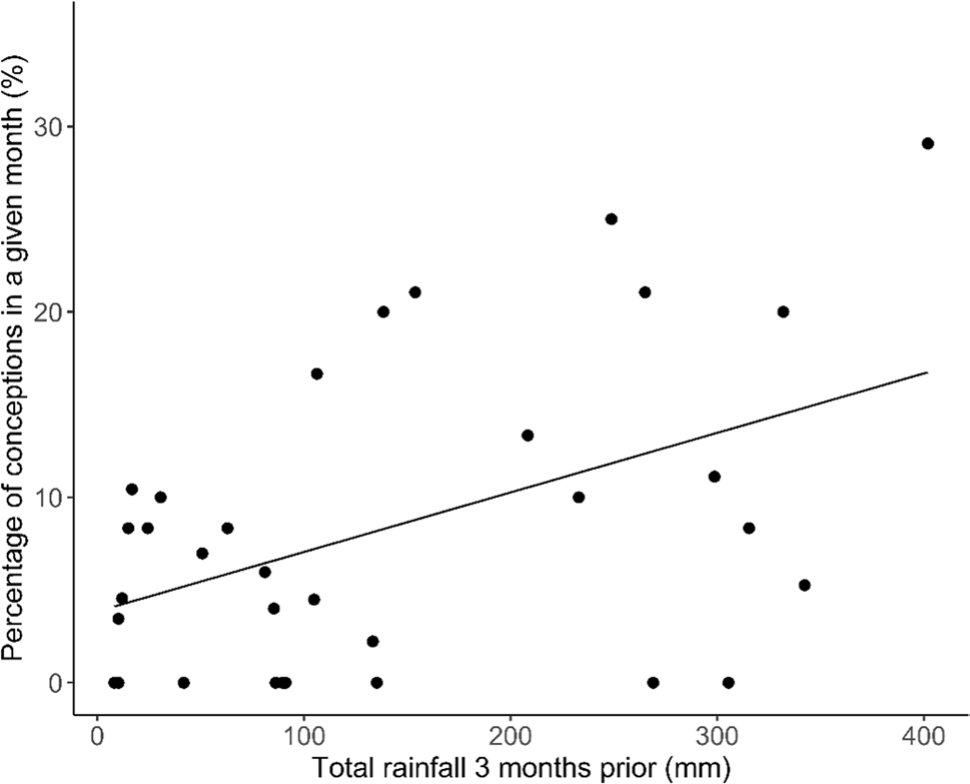
Relationship between the monthly percentage of conceptions in chital deer, and the rainfall 3 months prior to the month of conception

Conceptions can occur at any time of the year. However, conceptions were significantly seasonal with an average peak in the late summer (*p* < 0.05; Table 3; average = 3.1), 2.7 months before the peak in males in hard antler (Fig. 3). Despite the significant periodicity in conceptions, the peak of conceptions varied from year-to-year and approximately 5–20% of females that could have conceived did so in a given month (Fig. 3). Female conception rates were best explained by rainfall 3 months prior to conception, whereby increased rainfall correlated with increased conceptions (*R*^2^ = 0.299; Table 4; Fig. 4). As with the males, model selection resulted in two top model candidates (Supplementary Material Table 3), so model averaging was performed. Hard antler rate appeared in one of the top models (Supplementary Material 1 Table 3) with a marginally positive relationship with conception rates, but confidence intervals were broad following model averaging, and the proportion of variance explained was low. The relationship between rainfall and conception rates was consistent across years, based on a lack of support for models with interactions between year and rainfall metrics.

**Table 3:** Parameter estimates of cosinor model examining the seasonal patterns of the proportion of monthly conceptions (reproductively active) between 2013 and 2018

	Estimate	Std. error	*t* value	*p* value
(Intercept)	0.09	0.01	7.79	< 0.001
sinw	0.04	0.02	2.85	0.007

Significant sinw values indicate seasonal reproduction

**Table 4:** Model-averaged parameter estimates from the best (Δ AICc < 2) generalised linear models (GLM) from Supplementary Material Table 3 for the total number of conceptions per month with the total number of females that could have been pregnant in each month included as an offset

	Estimate	*z* value	2.50%	97.50%
Intercept	− 3.24	3.06	− 5.307	− 1.167
3 month	0.00	4.36	0.002	0.006
Hard antler	0.00	1.24	− 0.005	0.023
Daylight	− 0.10	0.63	− 0.403	0.208

## Discussion

In this study, both male and female chital deer reproduced year round, with seasonal peaks. Reproduction in females was strongly influenced by rainfall. In this population, females conceived after rain, which often coincided with summer (mid wet season) and autumn (late wet season). A large proportion of males were in hard antler at all times of year, but there was a discernible peak in the proportion of males in hard antler in mid-winter (the middle of the dry season). The highest proportion of conceptions did not coincide with the peak in hard antler in males, thus reproductive physiology was somewhat asynchronous in this population.

Male chital were more likely to be in hard antler during May to August which coincides with the shortest days of the year. Given that antlers take approximately 5 months to grow (Loudon and Curlewis 1988), longer daylengths were correlated with the initiation of antler growth. In their native range, the peak of hard antler is also seasonal, but it occurs during the summer, when days are longest (Moe and Wegge 1994; Sankar and Acharya 2004; Umapathy et al. 2007). This means that if daylength is a cue for the timing of antler development in male chital in Australia, they have switched their response from lengthening to shortening day lengths. The exact manner and time-frame when this switch occurred in the past 130 years is unknown. Three additional introduced chital populations have reported the timing of males in hard antler: Croatia, Hawai'i, and Texas (Graf and Nichols 1966; Howery et al. 1989; Kavcic et al. 2019). In Hawai'i and Texas, patterns are similar to those in native populations, but in Croatia, the male hard antler peak is from November-January, which is the opposite of all other northern hemisphere populations (Kavcic et al. 2019). It is unclear why chital in Australia and Croatia have reversed male reproductive seasonality. One clue could be the high proportion of males in hard antler outside the peak in both populations. On average, approximately 50% of males in Australia and Croatia were in hard antler during the months when the lowest proportion of males were in this antler phase, while the lowest monthly values in native populations were between 10 and 22% (Graf and Nichols 1966; Schaller 1967; Dinerstein 1980; Mishra 1982; Kavcic et al. 2019).

The patterns observed in Australia and Croatia suggest that some males start growing antlers in most, if not all, months of the year. This may also indicate that a breakdown in the physiological cues for reproduction occurred in these two introduced populations. This could have important implications for the mating system in these populations. Within the constraints of competitive ability, male chital have the potential to breed with females at any antler stage (Graf and Nichols 1966; Willard and Randel 2002), but testes size, and sperm volume and quality increase when they are in hard antler (Loudon and Curlewis 1988; Willard and Randel 2002). Given this change in their reproductive physiology, and the use of antlers in male contests, it is likely that there is a close association between reproductive success and antler stage. If we make the assumption that males in hard antler are able to outcompete other males, we can conclude that there are competitive males available to females year round, but that the proportion of males in hard antler relative to the number of receptive females will vary from year-to-year, depending on when females come into oestrus. As there are seasonal peaks in female reproduction, males should benefit from being in hard antler at approximately the time when most females come into oestrus. Because the exact timing of female conceptions shifts from year-to-year due to rainfall, there may be an advantage for males to vary the timing of antler growth to facilitate matings that will happen earlier or later than the average seasonal peak. This high variance in the timing of antler growth in Australia and Croatia may be a form of bet hedging.

Female reproduction, though occurring year round, was significantly seasonal in this population, and the timing of peaks in conception varied from year-to-year. Conceptions were positively correlated with the quantity of rainfall in the 3 months prior to reproduction. In deer species, females can conceive only when they are in the appropriate body condition (Mitchell and Lincoln 1973; Clutton-Brock et al. 1983; Flajsman et al. 2017; Paoli et al. 2018). Given the semi-arid climate in this region, it makes sense that females respond strongly to changes in rainfall. While rain during the wet season (December–March) accounts for almost 75% of the yearly rainfall, there is considerable year-to-year variation in the amount and timing (range 32–99%) varies tremendously from year-to-year. If seasonal rainfall patterns were predictable, the timing of births in this population would coincide with the end of the dry season, a period of low food availability. In addition, the season with highest food availability (late wet season) would coincide with the period of highest energetic need for lactating females, and for the survival of recently weaned fawns.

As the present study was not longitudinal, it was not possible to assess the timing of reproductive cycles for individual deer, which depends on the timing of sexual maturity, which in turn depends on the time of birth of the individual. In theory, factors that cause seasonal variation in juvenile mortality could also influence the current seasonal distribution of reproductive behaviour in populations that are not tightly synchronized by a photoperiodic trigger. To what extent the timing of antler formation and conception is affected by the timing of these events the previous year is unknown.

In this introduced population, the timing of reproductive activity i.e., conceptions and the peak of males in hard antler, is out of synchronicity by almost 3 months. In our population, reproductive asynchrony should lead to greater reproductive skew (variation in reproductive success among individual males), with fewer males available for mating when females are receptive (i.e., greater mismatch between available males and females potentially reduces the number of animals receptive at the same time; Garnier et al. 2001; Ostner et al. 2008; Sukmak et al. 2014). Female chital already exhibit a high degree of receptive flexibility, in that females may enter oestrus in any month of the year (Mylrea et al. 1999; Ahrestani et al. 2012). Some fawns born at any time of year may survive, and there may be some selection for males to be in hard antler throughout the year. Given the patterns we observed, we expect chital populations in Australia and Croatia to have high mating success and fawn survival outside the peak hard antler period compared to other populations, selecting most strongly for males to be in hard antler outside of the peak period. Data on fawn survival and success would be required from several populations to test this idea.

The pattern described here, in which reproductive asynchrony should lead to higher reproductive skew, would be different for other species with more discrete male reproductive seasonality. For example, in fallow (*Dama* *dama*) and roe deer (*Capreolus* *capreolus*), all males are in hard antler over a discrete period, outside of which no males are in hard antler and able to produce functionally competent spermatozoa (Gosch and Fischer 1989; Goeritz et al. 2003). Females coming into oestrus outside of this period of hard antler would not find a male producing functional spermatozoa. Selection pressure in these systems will be on males to respond to the same cues the females use to come into oestrus, or produce active sperm throughout the year. A change in external factors, that additionally affect the timing of female oestrus (e.g., rainfall), could have a deleterious effect on reproductive output in these populations.

Globally, in introduced ungulates, shifts in reproductive timing may cause reproductive asynchrony if the sexes are subject to different environmental cues for reproduction (Post and Forchhammer 2008; Moyes et al. 2011; Gaillard et al. 2013). Likewise, selection pressures that cause shifts in reproductive timing could impact a number of aspects of population biology, such as behaviour, mating patterns, and population genetics, which could lead to long-term consequences for population viability and may, in turn, enhance or impede attempts to control population sizes, depending on the goal of management.

## Supplementary Information

Below is the link to the electronic supplementary material.

Supplementary file1 (DOCX 27 KB)

Supplementary file2 (DOCX 90 KB)

## Data Availability

Data are being prepared for deposition into Dryad.
