# Supplementary material for: Dancing to a different tune: changing reproductive seasonality in an introduced chital deer population
Source: Oecologia. 2022 Aug 12;200(3-4):285–94. doi: 10.1007/s00442-022-05232-6 (PMC9675656; doi:10.1007/s00442-022-05232-6)
Supplement: Supplementary file 1 — Supplementary file1 (DOCX 27 KB) [file 442_2022_5232_MOESM1_ESM.docx]

Figure 1. Representation of the average monthly rainfall (orange) and average monthly day length (blue) in the Charters Towers region, North Queensland.

Table 1. Full set of models from AIC model selection for the monthly number of male chital deer in hard antler (presumed reproductively active) as the response variable, with the total number of stags sampled included as an offset, as compared with the environmental variables daylength, and 0, 1, 3, and 6 months rainfall prior to sampling including interaction with photoperiod, rainfall, and antler phase.

|  | df | logLik | AICc | ∆ AICc | weight |
| --- | --- | --- | --- | --- | --- |
| 6 month + daylength | 3 | -125.80 | 258.21 | 0.00 | 0.36 |
| 0 month + daylength | 3 | -126.01 | 258.62 | 0.41 | 0.29 |
| 1 month + daylength | 3 | -126.14 | 258.89 | 0.68 | 0.26 |
| 3 month + daylength | 3 | -127.65 | 261.91 | 3.70 | 0.06 |
| daylength | 2 | -129.34 | 262.96 | 4.76 | 0.03 |
| 6 month | 2 | -136.11 | 276.51 | 18.31 | 0.00 |
| 6 month * year | 10 | -130.36 | 287.40 | 29.19 | 0.00 |
| year | 5 | -145.55 | 302.69 | 44.48 | 0.00 |
| 3 month | 2 | -150.16 | 304.62 | 46.41 | 0.00 |
| 3 month * year | 10 | -140.45 | 307.57 | 49.36 | 0.00 |
| 1 month * year | 10 | -140.76 | 308.19 | 49.98 | 0.00 |
| 0 month * year | 10 | -140.98 | 308.63 | 50.42 | 0.00 |
| 1 month | 2 | -152.55 | 309.39 | 51.18 | 0.00 |
| 0 month | 2 | -153.16 | 310.61 | 52.40 | 0.00 |

Table 2. Summary of the top model from AIC model selection for the number of male chital deer in hard antler (presumed reproductively active) as the response variable, with the total number of observed males in a given month included as an offset, as compared with the environmental variables temperature, and 0, 1, 3, and 6 months rainfall prior to sampling including interaction with temperature, rainfall, and antler phase.

|  | Estimate | Std. Error | z-value |
| --- | --- | --- | --- |
| (Intercept) | 0.28 | 0.21 | 1.34 |
| temperature | -0.04 | 0.01 | -4.91 |
| 6 month | 0.00 | 0.00 | 4.46 |

Table 3. Full set of models from AIC model selection predicting the monthly number of conceptions (reproductively active) with the total number of females that could have conceived in a month included as an offset, as with covariates daylength, rainfall in the 0, 1, 3, and 6 months prior to conception including interaction with rainfall and year of conception.

| Model summary | df | logLik | AICc | ∆ AICc | weight |
| --- | --- | --- | --- | --- | --- |
| 3 month | 2 | -67.33 | 139.05 | 0.00 | 0.43 |
| 3 month + hard antler | 3 | -66.52 | 139.87 | 0.82 | 0.28 |
| 3 month + daylength | 3 | -67.11 | 141.05 | 2.00 | 0.16 |
| 3 month + daylength + hard antler | 4 | -66.44 | 142.30 | 3.25 | 0.08 |
| 3 month * year | 8 | -61.98 | 145.96 | 6.90 | 0.01 |
| 6 month + daylength | 3 | -69.92 | 146.68 | 7.62 | 0.01 |
| 6 month | 2 | -71.18 | 146.76 | 7.71 | 0.01 |
| 6 month * year | 8 | -62.53 | 147.05 | 8.00 | 0.01 |
| 6 month + hard antler | 3 | -70.56 | 147.96 | 8.90 | 0.00 |
| 6 month + daylength + hard antler | 4 | -69.92 | 149.27 | 10.22 | 0.00 |
| hard antler | 2 | -76.18 | 156.76 | 17.71 | 0.00 |
| 1 month | 2 | -76.26 | 156.92 | 17.87 | 0.00 |
| 1 month + hard antler | 3 | -75.15 | 157.13 | 18.08 | 0.00 |
| 0 month | 2 | -76.85 | 158.10 | 19.05 | 0.00 |
| daylength | 2 | -76.85 | 158.10 | 19.05 | 0.00 |
| 1 month + daylength | 3 | -75.69 | 158.21 | 19.16 | 0.00 |
| 0 month + hard antler | 3 | -75.92 | 158.66 | 19.61 | 0.00 |
| 0 month + daylength | 3 | -76.52 | 159.87 | 20.81 | 0.00 |
| 0 month + daylength + hard antler | 4 | -75.84 | 161.11 | 22.06 | 0.00 |
| year | 4 | -76.79 | 163.00 | 23.95 | 0.00 |
| 1 month * year | 8 | -71.64 | 165.27 | 26.22 | 0.00 |
| 0 month * year | 8 | -73.86 | 169.71 | 30.66 | 0.00 |
