# Supplementary material for: Dancing to a different tune: changing reproductive seasonality in an introduced chital deer population
Source: Oecologia. 2022 Aug 12;200(3-4):285–94. doi: 10.1007/s00442-022-05232-6 (PMC9675656; doi:10.1007/s00442-022-05232-6)
Supplement: Supplementary file 2 — Supplementary file2 (DOCX 90 KB) [file 442_2022_5232_MOESM2_ESM.docx]

**Detailed methods for stag antler identification from camera traps**

This camera trapping portion of this study was conducted at Spyglass Beef Research Facility, a cattle property covering 38,221 hectares in the Charters Towers district, Queensland, Australia (Figure 1). The study area contains grassland that consists of both native grasses, such as black speargrass (*Heteropogon contortus*) and kangaroo grass (*Themeda triandra*), and exotic grasses such as sabi grass (*Urochloa mosanbicensis*), red Natal grass (*Melinis repens*), and buffel grass (*Cenchrus ciliaris*). A variety of overstorey species include silverleaf box (*Eucalyptus pruinosis*), lancewood (*Acacia shirleyi*), bendee (*Acacia catenulata*) with yellowjacket (*Eucalyptus similis*), and ironbark (*Eucalyptus* spp.).

We used camera traps to determine the presence and relative number of chital in different habitats. To select camera trap locations, a grid with points 500m apart was created using ArcGIS (ESRI). To facilitate access to the locations, 124 points of the grid were selected that fell within 400m of a road or track. Bushnell Aggressor^TM^ cameras were placed at these locations for at least one month each between October 2017 and November 2018. Cameras were set to capture three images per trigger, with no delay between consecutive triggers. All images were stamped with the date and time. Cameras were installed approximately 30-50cm above the ground and pointed north or south to avoid the rising or setting sun. Vegetation in front of cameras was clipped to minimise interference and false triggers. Of the 124 cameras installed, 30 failed or the data they collected could not be analysed (e.g., they collected excessive false triggers). We therefore had photos from 94 operational cameras, representing 6707 trap days. Images were identified and organised using WildID and ZSL CTap software (Amin et al 2014; TEAM Network 2017). Chital stag antler stage (hard or velvet) was identified from images. Only images that could be positively identified were included in analyses. If a stag’s antler stage was uncertain or unknown, it was excluded.

For analysis, an “event” was defined as a sequence of photographs of one species that occurred following the previous sequence of a different species. When there were consecutive events of the same species, we ensured there was one hour or more between events (Bowkett et al. 2007; Amin et al. 2014; Rovero et al. 2017). This time frame was used to avoid repeated counting of the same individuals (Tobler et al. 2008; Rovero et al. 2017). We used Moran’s test in ArcGIS to compare the detection rates of chital across all cameras and found no significant spatial autocorrelation in our dataset (Moran’s index: 0.09, p = 0.313).


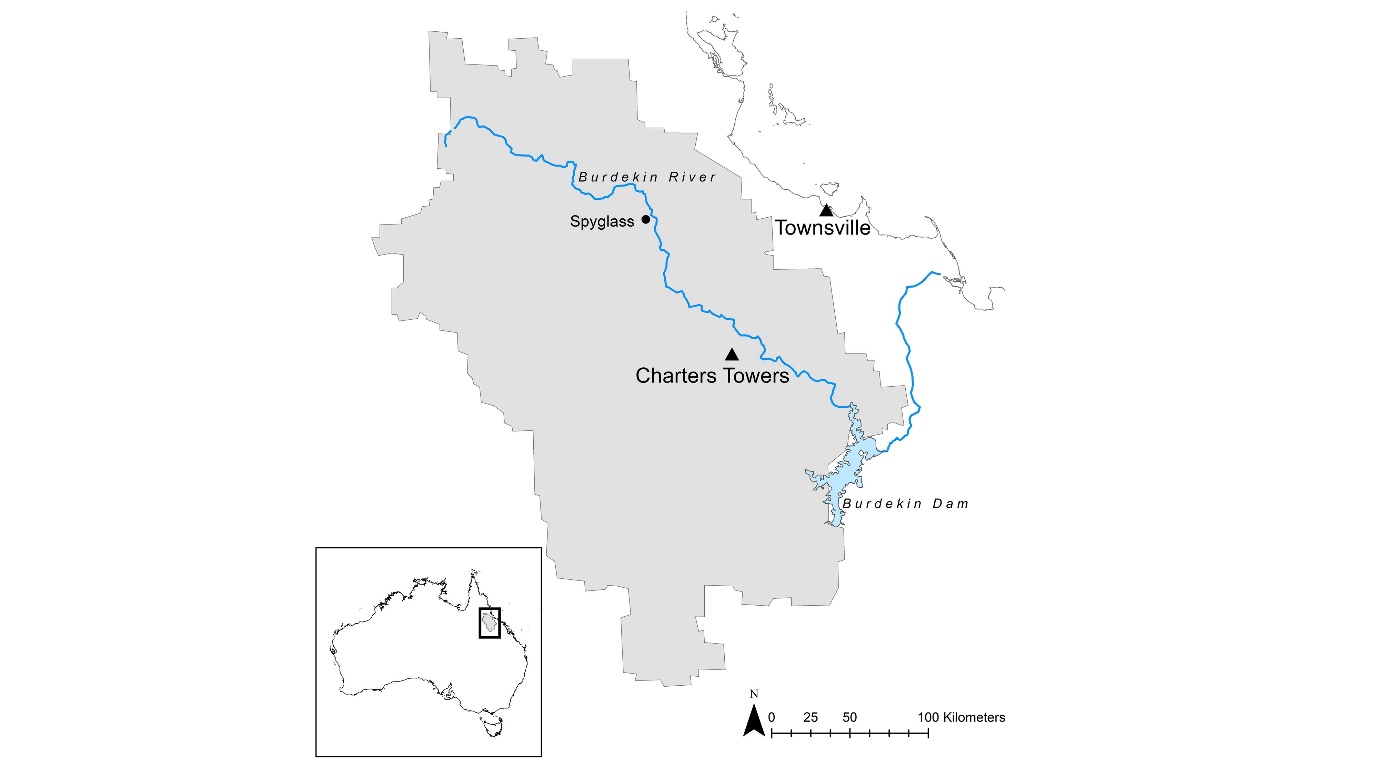


Figure 1. Location of Spyglass Beef Research Station in relation to the Charters Towers region (shaded grey; the location of the invasive chital population in North Queensland).
